# Timeliness and Quality of Response to Referrals Received by a Psychiatry Liaison Service for Older Adults During a Pandemic

**DOI:** 10.1192/bjo.2022.279

**Published:** 2022-06-20

**Authors:** Nirja Beehuspoteea, Robyn-Jenia Wilcha, Amelia Edwards, Agnes Mbazira

**Affiliations:** 1 South London and Maudsley NHS Foundation Trust, London, United Kingdom; 2 Croydon Health Services NHS Trust, Croydon, United Kingdom

## Abstract

**Aims:**

To improve timeliness of response and provide a committed plan to referrals received by the liaison service for older adults in Croydon University Hospital. Background: A quality improvement project in 2019 aimed to evaluate effectiveness of the liaison referral pathway. A questionnaire distributed to ward staff revealed some comments regarding ‘non-committal advice’ given by the liaison team.

**Methods:**

Data were collected from 44 referrals received by the liaison team in June 2021. Variables included referral date, reason for referral, date of first assessment, plan documented in the notes, date and details of committed plan of action.

Multi-disciplinary team (MDT) discussion identified that more committed advice could be provided by the following, which were implemented at the start of September 2021.

1. Huddle at the start of each day to triage and allocate referrals to appropriate members of MDT.
  - Prompt discussions with senior members of the team following assessment to discuss diagnosis and management.
  - Team teaching sessions were organised once a week, in the form of case-based discussions and role play, to improve communication skills, confidence and history-taking.

Data were then collected from 48 referrals received in September and October 2021.

**Results:**

Of the 44 patients in June, average time taken from point of referral to assessment was 1.27 days and to providing a concrete plan 1.80 days.

Of the 48 patients between end of September and October, average time to assessment was 1.31 days and to providing a concrete plan 1.88 days.

In June, 75% of patients were seen on same day or within one day and 50% had a concrete plan within one day.

In September/October, 65% of patients were seen on same day or within one day and 52% had a concrete plan within one day.

**Conclusion:**

These results highlight that assessments by older adult liaison service require detailed collateral history, investigations and MDT discussions.

While ‘obtain collateral history’ may not seem as committed a plan as prescribing medication, it remains an important part of old age psychiatry.

Given the rapid turnover of patients and increased pressures during the pandemic, it is the responsibility of the liaison team to communicate effectively with the wards and offer a timeline for completion of plan.

Following above changes, another questionnaire has been sent to request feedback on effectiveness of the liaison team.

